# Thrombolysis in an acute ischemic stroke patient with rivaroxaban anticoagulation: A case report

**DOI:** 10.1097/MD.0000000000014560

**Published:** 2019-02-22

**Authors:** Yen-Tung Chao, Chaur-Jong Hu, Lung Chan

**Affiliations:** a Department of Neurology, Taipei Medical University, Shuang Ho Hospital; b Department of Neurology, School of Medicine, College of Medicine; c Taipei Neuroscience Institute, Taipei Medical University, New Taipei City, Taiwan.

**Keywords:** ischemic stroke, novel oral anticoagulants, rivaroxaban, thrombolysis, tissue plasminogen activator

## Abstract

**Rationale:**

Whether intravenous recombinant tissue plasminogen activator (r-TPA) therapy can be administered in acute ischemic stroke patients treated with novel oral anticoagulants (NOACs), including rivaroxaban, remains controversial.

**Patient concerns:**

A 76-year-old woman with nonvalvular atrial fibrillation, who had been receiving 15 mg rivaroxaban once daily, was brought to the emergency department with right-side hemiparesis and aphasia. The onset of neurological deficits occurred 8 hours after the last dose of rivaroxaban administration.

**Diagnosis:**

The patient was diagnosed with acute ischemic stroke.

**Interventions:**

Intravenous infusion of 0.6 mg/kg of r-TPA (total dose: 29 mg) was performed 9 hours and 40 minutes after the last rivaroxaban administration. During r-TPA infusion, improvement in the patient's neurological deficit was observed.

**Outcomes:**

The clinical picture evidently improved from with National Institutes of Health Stroke Scale 21 to 16 on completion of r-TPA treatment.

**Lessons:**

Although current guidelines do not recommend administering thrombolytics in patients using NOACs with a doubtful anticoagulation status and administered within the last 24 or, even more strictly, 48 hours, this and other case studies suggest that r-TPA treatment could be considered in selected acute ischemic stroke patients receiving rivaroxaban or other Xa inhibitors, taking the patient's clinical condition and the prospective clinical benefits of r-TPA into account.

## 1 Introduction

Whether intravenous recombinant tissue plasminogen activator (r-TPA) therapy can be administered in acute ischemic stroke patients treated with rivaroxaban remains controversial.^[1–12]^ Approximately, 1% to 2% of patients with nonvalvular atrial fibrillation will experience an acute ischemic stroke despite receiving novel oral anticoagulants (NOACs).^[13]^ Thus far, r-TPA treatment has not been recommended for patients receiving rivaroxaban because of the increased risk of intracranial hemorrhage, in the absence of adequate drug elimination, and clearance or specific tests validating the lack of an anticoagulant effect.^[14,15]^ However, many of these patients will be critically evaluated at emergency rooms for eligibility for acute recanalization therapies including r-TPA and the use of intravenous thrombolysis could be safe under certain conditions. Withholding r-TPA therapy with no exception to all patients with acute ischemic stroke under rivaroxaban may also deny a considerable number of stroke patients an effective and innocuous treatment.

## 2 Case report

**Figure 1:**
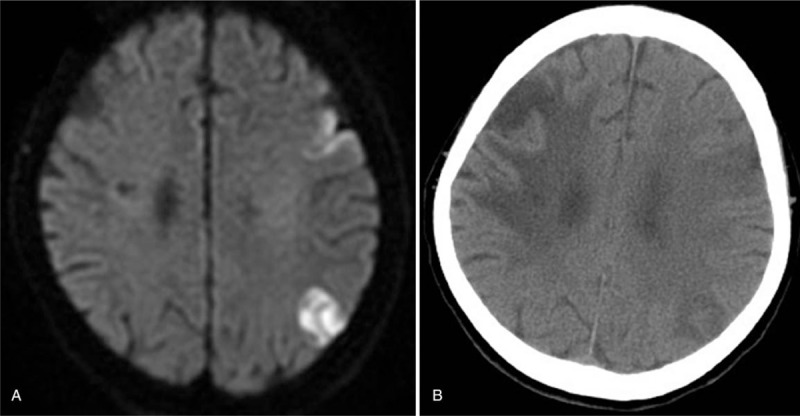
(A) Diffusion-weighted image. Hyperintense areas are observed in the left cortex and the posterior part of the middle cerebral arterial territory. (B) Computed tomography showing left cerebral infarction without hemorrhagic changes in the same site.

We report the case of a 76-year-old woman with a history of nonvalvular atrial fibrillation and hypertension, who had been receiving 15 mg rivaroxaban once daily since an episode of right middle cerebral arterial territory infarction 19 months earlier. Her CHADS_2_-VAS_c_ was 6. Adequate adherence to treatment was confirmed by her son. She arrived in the emergency department with an abrupt onset of consciousness disturbance, expressive aphasia, and right hemiparesis that occurred 30 minutes before the initial evaluation. The National Institutes of Health Stroke Scale (NIHSS) was 21. The onset of neurological deficits occurred 8 hours after the last dose of rivaroxaban administration. Clinical data on admission were as follows: blood pressure, 121/76 mmHg; prothrombin time (PT), 16.4 seconds (control: 11.0–14.5 seconds); international normalized ratio (INR), 1.41; activated partial thromboplastin time (aPTT), 137.0 seconds (normal: 32.0–45.1 seconds); thrombocyte count, 133 × 10^3^ mm^3^ (normal: 130 × 10^3^–400 × 10^3^ mm^3^); and creatinine level, 0.71 mg/dL, with an estimated glomerular filtration rate of 85.1 mL/min/1.73 m^2^. Electrocardiography revealed atrial fibrillation. Conventional brain noncontrast computed tomography (CT) showed encephalomalacia at the right fronto-temporo-parietal lobe due to a previous infarction of the right middle cerebral arterial territory. CT angiography revealed luminal narrowing of the right cavernous internal carotid artery, left cavernous internal carotid artery, and basilar artery. An acute ischemic stroke was diagnosed. We did not initiate endovascular intervention because her son did not agree to this invasive treatment, citing personal reasons. After considering the patient's clinical condition and the prospective clinical benefits of r-TPA, we decided to treat the patient in spite of the guideline recommending an at least 24-hour interval between rivaroxaban intake and thrombolysis. Intravenous infusion of 0.6 mg/kg of r-TPA (total dose: 29 mg) was thus performed 9 hours and 40 minutes after the last rivaroxaban administration, with informed consent. During r-TPA infusion, improvement in the patient's neurological deficit was observed (NIHSS score, 16 on completion of infusion), and her blood pressure and heart rate were adequately controlled. Brain magnetic resonance imaging (Fig. 1A) showed gyriform diffusion restriction in the left frontal, occipito-temporal, and parietal cortico-juxtacortical imaging series 16 hours after the onset of symptoms. A brain CT scan (Fig. 1B) performed 24 hours after r-TPA administration revealed no hemorrhagic change, with an NIHSS score of 14. After 4 weeks, the patient was discharged with an NIHSS score of 13 and remaining neurological sequelae of the right hemiparesis and motor aphasia.

## 3 Discussion

The safety and protocol for use of thrombolytic therapy after recent NOAC intake has still not been established. No randomized trials on the safety of r-TPA with rivaroxaban have been performed. Both the guidelines on thrombolysis and many studies insist on the necessity of normal coagulant tests as crucial criteria for r-TPA eligibility.^[16,17]^ However, conventional tests such as prothrombin time/international normalized ratio (PT/INR) and activated partial thromboplastin time (aPTT) are unreliable for measuring the anticoagulant effects of any NOACs.^[4,18]^ Although anti-Xa activity assays measure rivaroxaban concentration more precisely, they are not generally accessible, and their results may not be immediately available.^[19,20]^ Several other cases describing thrombolytic treatment in patients receiving rivaroxaban without hemorrhagic complications have been reported.^[1,6,8,21,22]^ The r-TPA dosages administered in such patients, including the standard protocol (0.9 mg/kg) and adjusted dose (0.6 mg/kg), are variably performed, with quite different outcomes.^[1,2,4–11]^ Fatal intraperitoneal hemorrhage with no clinical improvement was reported in a patient receiving r-TPA treatment at a dose of 0.6 mg/kg.^[2]^ Considering the prolonged aPTT test and uncertain anticoagulation status, we adopted a strategy of low-dose (0.6 mg/kg) r-TPA treatment. Nevertheless, one retrospective study indicated that intravenous r-TPA in NOAC patients (including 129 cases with rivaroxaban), as selected by their treating physician, is not associated with statistically significantly increased symptomatic intracranial hemorrhage risk (either serious systemic hemorrhage or r-TPA complication) compared with patients receiving warfarin or without an oral anticoagulant.^[23]^ Although rivaroxaban may favor hemorrhagic transformation, no correlation exists with the patient's clinical severity after intravenous thrombolysis.^[24]^ Moreover, one study on Wistar rats revealed downregulation of protease-activated receptor (PAR)-1 and PAR-2 expression in peri-ischemic lesions in the rivaroxaban group compared with the warfarin group. PAR-1 and PAR-2 play a role in modulating the effects of coagulation factors such as thrombin, factor Xa, and TPA and are associated with inflammation and neurodegeneration in stroke.^[25]^ This mechanism may also explain why, after induced cerebral artery occlusion and reperfusion with r-TPA, Wistar rats on rivaroxaban had a significantly lower intracranial hemorrhage volume.^[25]^

In our case, blood sampling was performed 9 hours and r-TPA administered 9 hours and 40 minutes after the last rivaroxaban intake, when the anticoagulant effect of rivaroxaban could be potent. Specifically, intravenous infusion of 0.6 mg/kg of r-TPA (total dose: 29 mg) was performed in a patient with extended aPTT. Considering at once the stroke severity, the expected clinical benefit, and the lack of alternative treatment, we explained the risks and benefits of this treatment to her relatives and performed it with their informed consent. No adverse events or intracranial hemorrhage occurred. The results in this case could imply that routine coagulation tests cannot precisely reflect plasma concentration or the anticoagulant effect of rivaroxaban mentioned in other studies.^[26]^ However, this may also have been only a special case, so that whether the same clinical improvement and outcome could be reproduced is debatable.

## 4 Conclusion

Little information is available regarding cases treated with intravenous r-TPA while receiving rivaroxaban. Although current guidelines do not recommend administering thrombolytics in patients using NOACs with a doubtful anticoagulation status and administered within the last 24 or, even more strictly, 48 hours, this and other case studies, although limited and debatable, suggest that low-dosage and, if possible, individualized r-TPA treatment could be considered in selected acute ischemic stroke patients receiving rivaroxaban or other Xa inhibitors. However, additional studies are necessary to establish the optimal treatment strategy and safety profiles of thrombolysis with r-TPA in stroke patients treated with NOACs.

## Acknowledgments

This manuscript was edited by Wallace Academic Editing.

## Author contributions

**Conceptualization:** Yen Tung Chao.

**Data curation:** Yen Tung Chao.

**Formal analysis:** Yen Tung Chao.

**Project administration:** Chaur-Jong Hu.

**Resources:** Yen Tung Chao.

**Software:** Yen Tung Chao.

**Supervision:** Lung Chan, Chaur-Jong Hu.

**Validation:** Lung Chan.

**Writing – original draft:** Yen Tung Chao.

**Writing – review & editing:** Yen Tung Chao, Lung Chan.

Yen Tung Chao orcid: 0000-0001-8812-6748.
